# 'Generation Pup' – protocol for a longitudinal study of dog behaviour and health

**DOI:** 10.1186/s12917-020-02730-8

**Published:** 2021-01-04

**Authors:** Jane Katherine Murray, Rachel Heather Kinsman, Michelle Susannah Lord, Rosa Elizabete Pinto Da Costa, Joshua Luke Woodward, Sara Cecylia Owczarczak-Garstecka, Séverine Tasker, Toby Grahame Knowles, Rachel Alison Casey

**Affiliations:** 1 Dogs Trust, London, UK; 2 Co-Evolve, Bristol, UK; 3 Linnaeus Group, Shirley, West Midlands UK; 4 Bristol Veterinary School, University of Bristol, Bristol, UK

**Keywords:** Canine, Dog, Puppy, Cohort, Longitudinal, Health, Behaviour, Genetic, Environmental, Management

## Abstract

**Background:**

Despite extensive research, many questions remain unanswered about common problems that impact dog welfare, particularly where there are multiple contributing factors that can occur months or years before the problem becomes apparent. The Generation Pup study is the first longitudinal study of dogs that recruits pure- and mixed-breed puppies, aiming to investigate the relative influence of environmental and genetic factors on a range of health and behaviour outcomes, (including separation related behaviour, aggression to familiar/unfamiliar people or dogs and obesity). This paper describes the study protocol in detail.

**Methods:**

Prior to commencing recruitment of puppies, the study infrastructure was developed, and subject specialists were consulted to inform data collection methodology. Questionnaire content and timepoint(s) for data collection for outcomes and potential predictors were chosen with the aim of providing the best opportunity of achieving the aims of the study, subject to time and funding constraints. Recruitment of puppies (< 16 weeks, or < 21 weeks of age if entering the United Kingdom or Republic of Ireland through quarantine) is underway. By 23 January 2020, 3726 puppies had been registered, with registration continuing until 10,000 puppies are recruited. Data collection encompasses owner-completed questionnaires issued at set timepoints throughout the dog’s life, covering aspects such as training, diet, exercise, canine behaviour, preventative health care, clinical signs and veterinary intervention. Owners can elect to submit additional data (health cards completed by veterinary professionals, canine biological samples) and/or provide consent for access to veterinary clinical notes. Incidence and breed associations will be calculated for conditions for which there is currently limited information (e.g. separation related behaviour). Multivariable statistical analysis will be conducted on a range of outcomes that occur throughout different life stages, with the aim of identifying modifiable risk factors that can be used to improve canine health and welfare.

**Discussion:**

The Generation Pup project is designed to identify associations between early-life environment, genotypic make-up and outcomes at different life stages. Modifiable risk factors can be used to improve canine health and welfare. Research collaboration with subject specialists is welcomed and already underway within the fields of orthopaedic research, epilepsy, epigenetics and canine impulsivity.

**Supplementary Information:**

The online version contains supplementary material available at 10.1186/s12917-020-02730-8.

## Introduction

Dogs are popular household pets in the United Kingdom (UK) with 24–30% of households estimated to own one or more dogs [1–3]. Health disorders of dogs have welfare implications for the dogs as well as financial and emotional implications for owners [4]. Behavioural disorders of dogs can negatively impact the welfare of dogs [5] and the strength of the human-animal bond [6], also increasing the risk of relinquishment [7] and euthanasia [8]. Many important health and behaviour conditions, including obesity, periodontal disease, aggression to dogs/people and separation related behaviour in dogs are influenced by multiple factors. Although hypothesised to be important in later-life outcomes, early-life management factors are under-studied for these conditions.

It has been estimated that 77% (95% CI: 62–92%) of the UK dog population are registered with a veterinary practice [3]. A study of 3884 dogs attending first opinion veterinary clinics in England between September 2009–March 2013 revealed that 75.8% of dogs had one or more health disorders diagnosed, the most prevalent of which were described as complex disorders (including otitis externa, periodontal disease, anal sac disorders, degenerative disease, diarrhoea, vomiting, obesity and heart murmur) [9]. Complex diseases such as these are likely to develop in affected dogs as a result of the interaction between multiple environmental, management and genetic factors. Detailed investigation of these factors within a large cohort of dogs enrolled as young puppies is needed to derive evidence-based results to better understand the various factors contributing to these disorders, and ways in which their incidence can be reduced in the future.

Canine behavioural problems are common and their development is also influenced by a complex interaction between environmental, experiential and genetic factors. Categorisation of presentations are also complicated by owner tolerance: in other words, numbers tend to reflect cases reported to be a ‘problem’ for owners, rather than those impacting on the welfare of the dog itself. A survey conducted in 2019 revealed that 76% of sampled dog owners living in the UK wanted to change one or more behaviours of their dog [10]. A sample of primary veterinary practices in England cited behaviour problems as the most common reason for death (including euthanasia) within dogs aged under 3 years (14.7% of deaths), and accounted for 4.2% of all deaths for dogs of any age (95% CI 2.0–8.0) [11]. Owners relinquishing dogs to rehoming centres often report problematic behaviour as the reason for relinquishment. For example, 34.2% of owners of a sample of 2806 dogs relinquished to the UK’s largest canine welfare charity (Dogs Trust) cited behavioural problems as the reason for relinquishment [7]. The precise extent to which behavioural problems are the main, or contributing factor, in an owner’s decision to relinquish a dog is currently unclear from published studies, as it is recognised that factors such as admission policies and the methodological details of data collection could introduce biases including social desirability bias [12]. Factors related to the owner, including ill health, expectations around dog ownership and house-moves are other factors that are also commonly cited as reasons for relinquishment [12].

In order to reduce the incidence of canine health and behaviour disorders, risk factor analyses are needed to identify causal factors that can be modified in the future. However, risk factor analyses of canine health and behaviour outcomes are often based on retrospective and cross-sectional studies (for example [13–15]). These studies often have limited information about environment or lifestyle [13], rely on owner memory to identify factors from earlier in life and can only identify associations rather than causal relationships between outcomes and potential risk factors [14]. Peer-reviewed research publications reporting work conducted at veterinary referral centres, can be subject to ‘referral bias’ [16, 17], whereas data collected from first opinion or primary care practices [18, 19] are limited to conditions presented to veterinarians. Thus, some disorders (e.g. vomiting or diarrhoea) that are frequently not presented to a veterinarian by owners [20], and other conditions such as being overweight/obesity that might not be routinely recorded in the veterinary clinical notes will be under-reported. In addition, behaviour problems, whether or not discussed in consultations, are rarely reported in electronic records. A longitudinal study of puppies that includes a variety of specific breeds and mixed-breed dogs is needed to provide additional and novel evidence-based information on which to base advice for stakeholders related to common canine health and behaviour conditions.

Prospective longitudinal studies offer many advantages over cross-sectional and retrospective studies. The relatively long lifespan of UK pet dogs, ranging from about 6 years to 16 years [11, 21, 22] contributes to the need for longitudinal studies so that we can better understand the effects of early-life experiences and management practices on health and behaviour outcomes in later life. The optimal approach is to undertake a longitudinal study, recruiting dogs as young puppies [23]. Such a study enables hypothesis testing of temporal relationships between previously suggested risk factors and owner- and/or veterinary-reported disorders through examining possible links with genetic and environmental exposure, including early-life exposures. Longitudinal studies of people have shown the ability of these studies to investigate a large number of health and behaviour outcomes at different ages, as demonstrated by the Avon Longitudinal Study of Parents and Children (ALSPAC) study which has more than 2000 peer-reviewed publications since 1990 [24]. Excluding dogs, but within the field of companion animal epidemiology, examples of longitudinal studies within the UK and Great Britain using owner-reported and veterinary-reported data have included investigation of mortality in geriatric horses [25], laminitis in horses/ponies [26], and health and behaviour outcomes in a birth cohort of pet cats [27]. Canine longitudinal studies in progress globally include three that focus on health, but not behaviour, outcomes. Two of these studies are breed-specific. The longitudinal study of Labrador Retriever dogs (‘Dogslife’) was launched in the UK in 2010 [28], from which early results on different aspects of health have been published [29, 30], whilst the ‘Golden Retriever Lifetime Study’ recruited 3000 Golden Retriever dogs (aged 6 months to 2 years) from the United States between 2012 and 2015 [31, 32], with analyses focusing primarily on cancer outcomes, but with other health-related analyses reported [33]. The ‘Dog Aging Project’ started recruiting dogs (of any age, breed or mixed breed) in the United States in 2019 and aims to recruit 10,000 dogs to the project, in order to study the genetic and environmental factors that affect aging and disease development [34].

This paper describes the design of the ‘Generation Pup’ longitudinal study that has been set up to collect data prospectively from owners and veterinarians (with owner permission) throughout the lifetime of enrolled dogs (pure-breed and mixed-breed dogs). The long-term aims of the Generation Pup study are to quantify the strength of associations between environmental exposures and genetic risk factors for specific canine health and behaviour problems of cohort dogs at different life stages, up to and including geriatric dogs, through the use of multivariable modelling techniques. Study results can be used by veterinarians, behaviourists, breeders, trainers and owners to improve canine health and welfare by basing management decisions on evidence-based recommendations.

## Methods/design

### Setting up the project

The core team of four researchers who were awarded a grant to fund the first 3 years of the study included two veterinarians with specialist diplomas in Veterinary Behavioural Medicine (R.A.C.), Animal Welfare, Ethics and Law (R.A.C.), Small Animal Medicine (S.T.), Veterinary Internal Medicine – Companion Animals (S.T.); a chartered Statistician (T.G.K.) and an Epidemiologist (J.K.M.). Following notification of the grant awarded to fund the first 3 years of the study (15th February 2015, with a project commencement date of 1st October 2015), this core study team started setting up the infrastructure of the project which included employing a web development company, recruiting a research officer (M.S.L) and a research assistant (R.H.K.).

The web development company was employed to set up the study website which includes a password-protected dashboard area for owners to access online study questionnaires, upload documents such as photos/scans of vaccination cards and access a diary feature to allow submission of additional free text data. The web development team convert questionnaires into an online format that involve personalisation related to the name of the dog and gender, logic within and between questionnaires that included avoiding unnecessary repetition of questions previously answered (for example, neuter status for dogs already reported to have been neutered). The online cloud-based survey system is programmed in Adobe Coldfusion and is hosted in a Microsoft Windows environment with survey responses stored in over 2600 SQL Server database tables. Personal data are encrypted using industry-standard strong cryptography.

The study team held a series of workshops during 2015, attended by veterinary and behaviour subject specialists employed at the University of Bristol. These workshops were used, together with available literature (for example, [9]) to prioritise topics for data collection, identify and discuss sources of data (e.g. owner-completed questionnaires, veterinary clinical notes, biological samples) and inform subsequent development and timing of survey questions. Workshop discussions included identifying outcomes of interest and potential risk factors for investigation, together with the age of dogs at which data should be collected – both for baseline data and to identify dogs with/without these specific outcomes. It was recognised that some outcomes (e.g. epilepsy) would require veterinary test results to confirm diagnosis, whilst other outcomes would be better classified from owner reports through the use of appropriate questions (e.g. separation-related behaviour). Since these initial workshops, subject specialists from other academic institutes (UK and non-UK) have been consulted and have generously provided advice and suggestions for questionnaire content.

Subject to availability, validated questions have been used in the Generation Pup questionnaires, for example the DORA questionnaire [35], whilst other validated questions have been adapted in consultation with the lead researcher of the original survey tool (for example, questions relating to cognitive function [36]). Where possible, data collected by owner-completed questionnaires will be tested for validity using appropriate comparative data which might include data obtained through veterinary clinical notes and video analysis. For example, a project is currently underway to validate dog mobility data from owner-reports using veterinary assessment and objective gait data (analyses of 3D limb motions and spatiotemporal gait parameters and force plate data).

Questionnaires have been, and continue to be, developed by the study team with input from subject specialists, as appropriate. Each new questionnaire is thoroughly checked by the web development team and the Generation Pup study team for routing logic, use of clear timescales for recall, clarity, non-specialist veterinary/behaviour terminology and non-ambiguous text, before test data are checked in the database prior to the questionnaire being launched.

Prior to recruiting the first puppies onto the study five questionnaires had been developed (three registration questionnaires, ‘Settling in’ and ‘12-weeks’). Thereafter, the study team develop questionnaires whilst the project is ongoing.

### Study population

Puppies of any pure-breed or mixed-breed (aged up to 16 weeks of age, or up to 21 weeks if they have entered the United Kingdom (UK)/Republic of Ireland (RoI) through quarantine), living in the UK or RoI can be registered on the study by their owners (aged 16 years or more).

### Recruitment

Registration of puppies began on 4 May 2016 when the study website (https://generationpup.ac.uk) was launched, whilst the principal investigator (RAC) and three co-investigators (TGK, JKM, ST) were employed at the University of Bristol (UoB). Between 4 May 2016 and 30 June 2017, the study was not widely advertised due to a pause in questionnaire development whilst RAC and JKM moved employment (from UoB to Dogs Trust); however, 200 dogs were registered during this time, with the date of birth of the oldest dog on the study recorded as 15 January 2016. These 200 dogs are referred to as the ‘early cohort’ (EC) at points within the text to help describe the methodology. Data from the EC are incorporated into the description of the cohort provided within this paper. Recruitment of dogs in the UK began in earnest in July 2017 and was extended to include dogs living in the RoI from 14 August 2018. Recruitment will continue until 10,000 puppies are registered with the study.

In order to minimise sampling bias, multiple methods of advertising are being used including display of posters and issue of flyers at veterinary practices and dog training venues, articles in veterinary/canine/other publications, social media and radio interviews. To date 26.1% (*n*=974) of 3726 participants had heard about the project through their veterinary practice, 18.6% (*n*=692) through a dog trainer, 14.4% (*n*=538) through social media, 11.9% (*n*=444) by word of mouth, 10.2% (*n*=381) from a magazine article, 5.7% (*n*=212) from the internet and 13.0% (*n*=485) learnt about it in another way (including via the breeder of the dog, from a rehoming centre or through television/radio promotion).

Owners may have up to five dogs registered on the study at any one time. By 23 January 2020, 3591 owners had registered a total of 3726 dogs on the study. Most (3476, 96.8%) had registered one dog, 101 (2.8%) had registered two dogs, 10 (0.3%) had registered 3 dogs, 2 (0.1%) had registered 4 dogs and 2 (0.1%) had registered 5 dogs on the study. A few puppies (including the 10 puppies registered by just two owners), have been registered by the breeder or rehoming centre prior to being sold/rehomed. Dogs that change ownership/carer can continue their involvement in the study if their new owner/carer wishes to participate in the study.

### Data collection

As part of the registration process, participants provide consent for questionnaires to be issued at set time points and for data to be stored. Additional, and optional, consent can be provided by participants for one or more of the following aspects which are outlined in more detail later in this section:

- veterinary health cards (oral health, body condition score, assessment of heart)
- owner-submitted, non-invasive canine biological samples (hair brushings, urine, faeces, skin swab, buccal swab)
- access to the dog’s veterinary clinical notes
- contact about additional research studies linked to ‘Generation Pup’. (For example, filming aspects of their dog’s behaviour.)

The owner’s online dashboard includes a ‘diary’ section where owners can add free text information relating to any events they would like to report, under categories including ‘A trip to the vet’, ‘A new experience’, and since April 2020 ‘COVID experiences’. Diary data can be entered at any timepoint and on multiple occasions.

### Owner-completed questionnaires

All questionnaires are self-administered, and participants can choose to complete questionnaires online or via paper copies. Most owners complete online questionnaires. By February 2020, only 71 (1.9%) owners were completing paper questionnaires, 61 of which started completing online questionnaires and subsequently switched to paper questionnaires.

Each questionnaire is split into 2–19 different sections (‘steps’) with each focussing on a different topic area (for example ‘diet’, ‘exercise’, ‘health’) (Additional file 1 contains details on topics covered within each questionnaire and links to PDF versions). Most questions are ‘closed’ questions, with free text boxes available to capture responses that are not covered in the options available to the owner. Estimated completion time ranges from 20 to 30 min for most questionnaires, depending on the owner’s responses. Owners can submit each step on completion and return to finish the remaining steps at a later date, subject to the expiry date (detailed elsewhere in this section). The study team made the decision to split each questionnaire into discrete steps, so that owners could, if they wished, complete a questionnaire over a few days, hence enabling our questionnaires to take longer to complete than the optimal time of 20 min [37].

Owners complete two questionnaires (‘about me’ (AM) and ‘about my household’ (AMH)) before registering a puppy with the study by completing the ‘about my puppy’ (AMP) questionnaire. Early-life (< 16 weeks) data are collected in 1–3 questionnaires, depending on the age of the puppy at the time of registration, as follows:

- ‘Settling In’ (SI) questionnaire: completed 1–3 weeks after acquisition of the puppy, or until 12 weeks of age, whichever is sooner.
- ‘12 weeks’ (12w) questionnaire: available for completion from age 12 weeks (84 days) until 15.5 weeks (108 days).
- ‘16 weeks’ (16w) questionnaire: available for completion from age 16 weeks (112 days) until 19.5 weeks (136 days).
- SI/12w combined questionnaire: issued to puppies registered between age 12 weeks (84 days) and 15.5 weeks (108 days).
- SI/12w/16w combined questionnaire: issued to puppies registered between 15.5 weeks (109 days) and 16 weeks (112 days).

Details regarding timing and availability of subsequent questionnaires are provided in Table 1. Questionnaires are issued more frequently when dogs are young (Table 1), to reduce the likelihood of recall bias impacting the accuracy of data related to early-life experiences and management practices reported by owners [38]. These early-life experiences are hypothesised to be important for many outcomes that will be investigated in the future (including noise phobia, aggression to people/dogs and mobility problems). The timing and content of study questionnaires has been based on a subjective balance of optimising collection of data relating to outcomes of interest and potential risk factors at appropriate timepoints, whilst minimising the time that owners need to dedicate to the study. It should be noted that in order to study outcomes related to dog behaviour, detailed questions about the dog’s behaviour in a variety of different contexts are required. For example, separation-related behaviour (SRB) should be classified on the dog’s behaviour when left alone (and in comparison to the dog’s behaviour when not left alone), rather than on the owner’s perception of the dog having SRB problems. However, perception of a problem is also of interest as a potential predictor for relinquishment and/or aversive training methods being introduced. This example of SRB illustrates the need for detailed questioning in order to classify dogs correctly with respect to this outcome.

**Table 1:** Online questionnaire availability and biological sample request schedule for owners of Generation Pup study dogs

Questionnaire	Available	Duration ofavailability (days)	Questionniarelaunch date	Biological Samples requested^**a**^(Date sampling commenced)
**Settling In (SI)**	1 week after acquisition	14 (max)	06/05/16	
**12 weeks (12 w)**	12 w (84 days)	24	06/05/16	12–16 weeks: F, U, H, BS (August 2017)
**16 weeks (16 w)**	16 w (112 days)	24	19/07/17	
**5 months (5 m)**	5 m (150 days)	21	06/08/18	
**6 months (6 m)**	6 m (180 days)	24	26/10/17	F, SS (April 2019)
**7 months (7 m)**	7 m (210 days)	28	19/11/17	
**9 months (9 m)**	9 m (274 days)	42	10/02/18	
**12 months (12 m)**	12 m (365 days)	42	16/04/18	F, U, H, BS, SS (November 2018)
**15 months (15 m)**	15 m (456 days)	42	17/07/18	
**18 months (18 m)**	18 m (547 days)	42	19/10/18	
**2 year (2 y)**	2 y (730 days)	42	09/04/19	F, U, H, SS (March 2019)
**2.5 year (2.5 y)**	2.5 y (912 days)	42	06/11/19	
**3 year (3 y)**	3 y (1095 days)	42	09/04/19	F, U, H, SS (March 2019)
**3.5 year (3.5 y)**	3.5 y (1277 days)	42	06/11/19	
**4 year (4 y)**	4 y (1460 days)	42	05/03/20	F, U, H, SS (January 2020)

^a^*F* faecal sample, *U* urine sample, *H* hair sample, *BS* Buccal swab, *SS* Skin swab

Discussions with a canine orthopaedic specialist revealed that baseline mobility data were needed from young dogs, which prompted the addition of a 5 m survey. The 5 m survey was introduced more than 9 months after the launch of the 6 m survey. The 5 m survey was given a shorter availability timeframe (21 days) in comparison to the 16 w and 6 m surveys, in order that a minimum of 7 days would elapse before an owner was asked to complete the 6 m survey. Ideally, the 16 w, 5 m and 6 m surveys would have had the same duration of availability (21 days), but availability of the 16 w and 6 m surveys were not amended as the project was already underway. At this stage it is anticipated that questionnaires will be issued for the duration of the lives of the dogs; however, this will be reassessed based on questionnaire completion data available for dogs aged 12 years. Owners are sent emails notifying them when a questionnaire becomes available for completion and informing them of the questionnaire expiration date. If the questionnaire has not been started within 10 days, then a reminder email is sent (6:00 PM), with a SMS text message reminder following at 7:00 PM (for those who have still not started their questionnaire, if the owner has provided consent to receive text messages, *n *= 2447, 65.7% of owners). Owners who start, but do not complete their questionnaire are sent a reminder email 7 days after they last submitted questionnaire data. Owners electing to complete paper questionnaires are mailed a copy of the questionnaire at the appropriate timepoint, with no reminders issued.

#### The ‘early cohort’

Early challenges in project administration that have been previously described, resulted in some questionnaires (16 w-2.5 y) not being developed in time for completion by owners of the ‘early cohort’ (*n *= 200), i.e. dogs born prior to April 2017. The three registration questionnaires (AM, AMH, AMP) and the SI and 12 w questionnaires were available to the ‘early cohort’ (EC), before a gap in age-specific questionnaires (16 w-2.5 y), until questionnaire development had ‘caught up’ with these dogs at age 3 years. During this time, two additional surveys were issued to EC owners, with the aim of maintaining engagement and contact with these owners, in addition to collecting data on important early-life events and management practices. The first was titled the ‘Catch up Survey’ (Additional file 1), which was issued to owners of 164 EC dogs in September 2017 with an explanation about the delayed standard questionnaire development. The ‘Catch up Survey’ was completed for 63/164 dogs (38.4%). The second was a copy of the 12 m questionnaire which was named ‘Spring 2018 Survey’, and issued to owners of EC dogs who were still registered with the study, and aged > 408 days (*n *= 195) on 16 April 2018, as they were too old for completion of the standard 12 m questionnaire. The ‘Spring 2018 Survey’ was completed for 50/195 dogs (25.6%).

Of 200 EC dogs, 15 (7.5%) had been withdrawn by 22 January 2020 from the study by their owners, and the 3 y questionnaire had been completed by 36.8% (68/185) of owners of the remaining EC dogs.

#### Health scores

Step 7 of the 15-month questionnaire contains questions relating to various health score tests, including hip/elbow dysplasia, eye and hearing tests. Following completion of this step, or expiry of the 15 m questionnaire if the owner does not complete this questionnaire, this set of questions is available on the owner’s dashboard for updates, if for example new test data becomes available when the dog is older.

### Oral health (OH) cards, heart murmur (HM) cards and body condition score (BCS) cards

The OH and HM cards were developed by a team of veterinarians including specialists in companion animal internal medicine (OH and HM) and cardiology (HM). The BCS card used a standardised nine-point scoring system available from Royal Canin®, based on a validated tool [39]. Since September 2017, owners consenting to receiving ‘veterinary health cards’ (OH, HM, BCS cards) are mailed these cards and a pre-paid return envelope when their puppies are initially registered at age < 16 weeks (HM and BCS), at age 1 year (OH, HM, BCS) and annually thereafter (OH, HM, BCS). Copies of cards are provided in Additional file 2. Owners are requested to take these cards with them to a routine puppy consultation (HM and BCS only), at their dog’s annual vaccination and health check, or to another convenient consultation that the owner has booked for other reasons. Puppy owners are informed that ideally the HM card should be completed at age 8–16 weeks and the BCS card at age 4–7 months. Cards can be completed by a veterinarian (OH, BCS, HM) or veterinary nurse (OH and BCS), before being returned by the dog’s owner to the Generation Pup study team. Emails reminding owners to complete these cards are scheduled to coincide with likely booster vaccination dates, based on the dog’s age, for example 1 year 3 months, 2 years 3 months, and so on.

### Canine biological samples

Owners who consent to participate in the provision of non-invasive, canine biological samples for the study are sent sampling packs containing detailed instructions on how to take the samples (including links to online instructional videos), the equipment needed (e.g. faecal collection pots, gloves), and a pre-paid return envelope. Owners are not offered the results of any tests and are informed that the samples will be stored and analysed to help identify risk factors for diseases that occur later in life. Samples are sent to the Clinical Investigation Centre at the Royal Veterinary College, where they are processed and stored on a short-term basis before being transported to a biobanking company for long-term storage. Sampling packs are sent out when dogs are aged 12–16 weeks, 6 months, 12 months and then annually, according to the schedule presented in Table 1. Current plans include collecting faecal, urine and hair samples annually from age 5 years. Canine biological samples are not requested from owners living in the RoI, due to postal restrictions. Examples of current plans for the samples include genetic analysis of DNA (buccal swabs), measuring cortisol and quantifying exposure to nicotine (hair samples), parasitic load (faecal samples), analysis of metabolomic profiles (urine samples) and microbiome analysis (faecal samples and skin swabs).

### Veterinary clinical notes

Owners indicating at registration or subsequently that they are happy to provide consent for access to their dog’s veterinary clinical notes, are asked to sign and return a consent form that can be shared with their veterinary practice as proof of consent. Processes are in place requesting that owners check that their consent forms are up-to-date and new forms are completed if needed (e.g. change of owner address or change of veterinary practice), or if more than 2 years have elapsed since the original/previous form was signed. Obtaining clinical notes from veterinary practices was piloted in November–December 2019 using a sample of 53 dogs; clinical notes were provided for 75.5% (*n*=40) of the dogs. Clinical notes are securely stored prior to free text data extraction using customised queries written in languages such as Python or R.

### Retention of owners

A variety of engagement strategies are utilised with the aim of maximising retention of owners in the study and thus their provision of data. These engagement strategies include social media sites (Facebook and Instagram), monthly ‘dog of the month’ and ‘owner of the month’ prize draws (with an estimated annual cost of £120), competitions to win prizes donated by other companies (for example, a rucksack) and a 5% discount off a holiday after completing the ‘Spring 2018 Survey’, 7 m and/or 18 m surveys (donated by a company offering dog-friendly cottage holidays, for surveys completed between October 2018 and July 2020). Monthly Infographics of ‘fun facts’ are posted on social media sites and on the Results tab of the Generation Pup website (https://generationpup.ac.uk/results). Owners are issued with a ‘contribution to animal welfare’ certificate each time their dog is included in research that is presented at a scientific conference or published in a peer-reviewed journal. An element of gamification links the number of certificates awarded to the ‘title’ awarded to the dog. For example, a dog is awarded a status of ‘Graduate’ based on being awarded four certificates for data contributing to four research articles, and a status of ‘Dogtor’ for seven certificates, whereas 25 certificates earns the dog the status of ‘Pawfessor’. By March 2020 seven versions of the certificates had been issued, 94 dogs had been awarded the maximum to date of seven certificates, with 1691 dogs receiving at least one certificate.

### Online dashboard

Questionnaires available for completion, the certificates, diary feature, and a gallery of photographs of the owner’s dog that have been uploaded are available to owners through a password-protected personal online dashboard. A cartoon dog appears throughout the questionnaires to add an element of fun to questionnaire completion, whilst emails are personalised for the owner. Once the dog has reached 6 months of age, each owner is able to download from their dashboard a ‘Doggy Dossier’ of facts and photographs that they have provided to date. The ‘Doggy Dossier’ provides the owner with an attractive memento of the early months of their dog’s life and is currently prepopulated with information until the dog reaches 12 months of age, with plans to extend this feature.

### Statistical analysis and power calculations

The incidence of specific health and behaviour outcomes will be calculated, and breed differences assessed subject to sufficient sample size.

Power calculations will vary according to the outcome of interest, taking into account the age of dogs at the time of assessing data for presence/absence of the outcome, the estimated sample size and prevalence within our dataset. Detailed power calculations based on current questionnaire completion rates and loss to follow up rates, are provided in Additional file 3. For example, we estimate that the study will have at least 80% power to detect an Odds Ratio (OR) > 1.75 (for risk factors that at least 20% of controls are exposed to) for outcomes at age 5 years with a prevalence of > 7.2% (e.g. conservative prevalence estimates for aggression to unfamiliar dogs/people, based on available research [14, 40]). Long-term follow up of the study population will enable study of mid and later life outcome measures of importance to canine welfare (e.g. dental disease, obesity, cognitive dysfunction, osteoarthritis, heart failure, survival). For example, prevalence estimates of canine cognitive dysfunction syndrome (CDS) in dogs > 8 years of age range from 14 to 60% [41–44]. Conservatively, assuming a CDS prevalence of 14% at age 8 years, we estimate having 80% power to detect OR> 1.50 for variables that 20–30% of controls are exposed to (Additional file 3).

Statistical analysis on a range of health and behaviour outcomes will be conducted using multivariable modelling techniques to identify and quantify the independent effects of novel genetic and environmental risk factors for outcomes. Clustering at the household level will be accounted for in the analysis using multilevel models, or where the sample size does not justify this approach, by randomly selecting one dog per household for inclusion in the dataset for analysis. Knowledge gained from these analyses can then be used to inform interventions which aim to improve canine welfare in the future.

## Results

### The cohort profile

The cohort profile presented here is based on dogs registered with the Generation Pup study by their owners between May 2016 and January 2020, (*n*=3726).

Most dogs that are registered on the study were intended by owners (at registration) to be kept as family pets/company for the owner (3674, 98.6%), with some of these dogs also being used as working/assistance dogs and/or for activities such as showing and breeding. Of the 53 dogs (1.4%) that were not intended as family pets/company for the owner, some dogs had multiple intended purposes with 21 destined to be working dogs with one or more ‘jobs’ (3 assistance dogs, 10 gundogs, 7 sheep/cattle dogs, 1 search and rescue dog, 1 guard dog), 22 to be used for competitions and 2 for breeding. Five dogs were currently still at a rehoming centre and owners of two dogs recorded no specified intentions for the dog (pet, companion or other purpose).

Summary data about the dogs, respondents and their households are provided in Table 2.

**Table 2:** Summary owner-reported dog, respondent and household data for dogs registered with the Generation Pup study

Variable. (Number of responses from 3726 registered dogs)	Categories	Number of dogs	Percentage based on available responses
**Dog demographic data**			
**Sex of dog (*n = *3726)**	Male	1891	50.8
	Female	1835	49.3
**Source of dog**^**a**^ **(*n *= 3721)**	A hobby or occasional breeder	1800	48.4
	A Kennel Club (KC) assured breeder	824	22.1
	A professional breeder	625	16.8
	A charity/rescue/adoption/rehoming organisation	197	5.3
	Homebred	141	3.8
	Breeder –type (hobby/KC/professional) unknown (e.g. ‘online’ sale, family/friend).	101	2.7
	Breeder/origin unknown (not bought directly from breeder, eg stray, rehomed from an owner who no longer wanted the puppy)	33	0.9
Owner-reported neuter status at time of 12 m survey completion (aged 365–407 days) *n *= 1104 (exc 218 missing data)	Not neutered	655	59.3
	No, but chemically castrated within the last 3 months	5	0.5
	No, but chemcially castrated more than 3 months ago	1	0.1
	Neutered before age 9 months	282	25.5
	Neutered since age 9 months	161	14.6
**Respondent demographic data (at registration)**			
**Gender of respondent (*n *= 3719)**	Male	381	10.2
	Female	3338	89.8
**Age of respondent (*n *= 3718)**	16–24 years	317	8.5
	25–34 years	853	22.9
	35–44 years	838	22.5
	45–54 years	832	22.4
	55–64 years	632	17.0
	65+ years	246	6.6
**Employment status of respondent (*n *= 3685)**	Employed	2160	58.6
	Self-employed	468	12.7
	Retired/Pensioner	483	13.1
	Homemaker/housewife/househusband	282	7.7
	Currently not working	155	4.2
	Student	137	3.7
**Employment sector: employed/self-employed respondents (*n = *2628)**	Work with animals	405	15.4
	Do not work with animals	2223	84.6
**Respondent works with dogs (*n *= 2628)**	Yes	371	14.1
	No	2257	85.9
**Previous dog ownership (*n = *3726)**	I have almost always had a dog/s - but with breaks for other life circumstances	638	17.1
	I have always owned a dog/s since having a family dog/s as a child	860	23.1
	I have had other dogs during my adult life	1103	29.6
	This is my first dog	510	13.7
	We had a family dog when I was a child, but I haven’t had one again until now	609	16.3
	Other responses that could not be assigned into any of the above categories	6	0.2
**Household demographic data (at registration)**			
**Geographical location (*n = *3726)**	England	3054	82.0
	Wales	173	4.6
	Scotland	336	9.0
	Northern Ireland	56	1.5
	Republic of Ireland	107	2.9
**Urban/rural location, as described by the respondent (*n *= 3706)**	City or urban area	605	16.3
	Suburban area	971	26.1
	Village or small town	1395	37.5
	Rural area (for example, small village or hamlet)	623	16.7
	In a remote/isolated area	112	3.0
**Tenure (*n *= 3725)**	Own home	3028	81.3
	Rent home	697	18.7
**Children (aged** **<** **17 years) in the household (*n = *3726)**	None	2572	69.0
	One or more	907	24.3
	Data not provided	247	6.6
**Number of adults in the household (*n = *3726)**	One	431	11.6
	Two or more	3048	81.8
	Data not provided	247	6.6
**Household composition (*n = *3479)**	One adult, no children	356	10.2
	Two or more adults, no children	2216	63.7
	One adult, one or more children	75	2.2
	Two or more adults, one or more children	832	23.9
**Annual household income (*n *= 3120)**	<£15,000	192	6.2
	£15,000–£24,999	394	12.6
	£25,000–£34,999	465	14.9
	£35,000–£44,999	509	16.3
	£45,000–£54,999	434	13.9
	>£55,000	1126	36.1
**Highest level of qualification achieved within the household (*n = *3725)**	No formal qualifications	50	1.3
	GCSEs / O' levels or equivalent	353	9.5
	A’ levels or equivalent	478	12.8
	Undergraduate degree	1141	30.6
	Postgraduate degree	1119	30.0
	Vocational qualification (for example NVQ)	149	4.0
	Higher qualification (for example HND)	435	11.7
**Number of dogs in the household (*n = *3725)** *Median=1, Interquartile range 1–2.*	One	2179	58.5
	Two	885	23.8
	Three	363	9.7
	Four	123	3.3
	Five	65	1.7
	Six	36	1.0
	Seven	28	0.8
	8–13	46	1.2
**Cat(s) in the household (*n = *3725)**	No	2687	72.1
	Yes	1038	27.9

^a^Definitions were not provided to owners relating to the categories provided for the source of the dog

To date, 65.6% (2446/3726) of the cohort are described by their owners as being a ‘specific named breed’, compared with 935 (25.1%) that are a ‘cross of two specific breeds’. The most common cross breeds within our dataset are currently Cocker Spaniel/Poodle (also known as ‘Cockerpoo’) dogs, *n*=242; Springer Spaniel/Cocker Spaniel (‘Sprocker’) dogs, *n*=76; and Labrador/Poodle (‘Labradoodle’) dogs, *n*=65. Within our dataset, 197 (5.3%) were described by their owners as a ‘mixed breed without known parentage, but of a particular type, e.g. Collie type or collie Cross)’, whilst 148 (4.0%) were described as ‘a mixture of different/unknown breeds’. To examine the extent to which the cohort is representative of dog breeds in the UK, the proportion of each breed within the ‘specific named breeds’ category was compared with the Kennel Club Breed Registration Statistics databases 2018–2019 (Kennel Club, 2019). Dogs registered with the UK Kennel Club in 2018–2019 were used for comparison with Generation Pup ‘specific breed’ dogs, most of which (70.4%, 1723/2446) were born during 2018 and 2019. For this comparison, the number of each breed registered on the Generation Pup study was calculated as a proportion of the ‘specific breed’ dogs registered (*n*=2446). Two-tailed z-tests were used to assess whether breed proportions for the 22 different breeds listed in Table 3 were significantly different between the two populations and the Bonferonni correction was used to account for multiple testing, hence significance was set at *P*< 0.002. No significant difference in proportions was evident for most (*n*=15) breeds. However, French Bulldogs, Pugs and Bulldogs are currently under-represented within our dataset (*P*< 0.0001), whilst Border Collies, Golden Retrievers, Jack Russell Terriers and Flat Coated Retrievers are over-represented (*P*< 0.0001) (Table 3).

**Table 3:** The 20 most common breeds registered with ‘Generation Pup’ and the UK Kennel Club (2018–2019)

Breeds most frequently registered with the Generation Pup study (of those stated to be of a specific breed, ***n=***2446)			Breeds most often registered by the UK Kennel Club (mean data for 2018–2019, ***n=***241,583/year)		
Breed	N	% of all dogs of a specific breed	Breed	N	% of all registered breeds
***20 most common breeds (in descending order)***			***20 most common breeds (in descending order)***		
Retriever (Labrador)	368	15.04	Retriever (Labrador)	35,937	14.88
Spaniel (Cocker)	270	11.04	French Bulldog	35,223	14.58
Border Collie^a^	207	8.50	Spaniel (Cocker)	22,795	9.44
Retriever (Golden)^a^	119	4.87	Bulldog	10,297	4.26
German Shepherd Dog	97	3.97	Spaniel (English Springer)	9395	3.89
Spaniel (English Springer)	87	3.56	Pug	8247	3.41
Dachshund (all types combined: Miniature and Standard, Long/Smooth/Wire Haired)	70	2.86	Retriever (Golden)	8108	3.36
Border Terrier	64	2.62	German Shepherd Dog	7068	2.93
Jack Russell Terrier^a^	57	2.33	Dachshund (all types combined: Miniature and Standard, Long/Smooth/Wire Haired)	6701	2.77
Miniature Schnauzer	53	2.17	Miniature Schnauzer	5259	2.18
Cavalier King Charles Spaniel	42	1.72	Border Terrier	4799	1.99
Chihuahua (Long/Smooth coat)	34	1.39	Staffordshire Bull Terrier	4656	1.93
Staffordshire Bull Terrier	33	1.35	Chihuahua (Long/Smooth coat)	3546	1.47
Whippet	33	1.35	Cavalier King Charles Spaniel	3477	1.44
French Bulldog^b^	32	1.31	Boxer	3368	1.39
Flat Coated Retriever^a^	31	1.27	Whippet	3311	1.37
Hungarian Vizsla	30	1.23	Hungarian Vizsla	2787	1.15
Beagle	28	1.14	Shih Tzu	2418	1.00
Shih Tzu	27	1.10	Beagle	2136	0.88
Boxer	23	0.94	Border Collie	2071	0.86
***Other breeds, for comparison with top 20 breeds within the Kennel Club dataset***			***Other breeds, for comparison with top 20 breeds within the Generation Pup cohort***		
Bulldog^b^	21	0.86	Jack Russell Terrier	236	0.10
Pug^b^	21	0.86	Flat Coated Retriever	1159	0.48

^a^Breeds currently over-represented within the Generation Pup cohort (*P*< 0.0001)

^b^Breeds currently under-represented within the Generation Pup cohort (*P*< 0.0001)

### Response rates

**Fig. 1:**
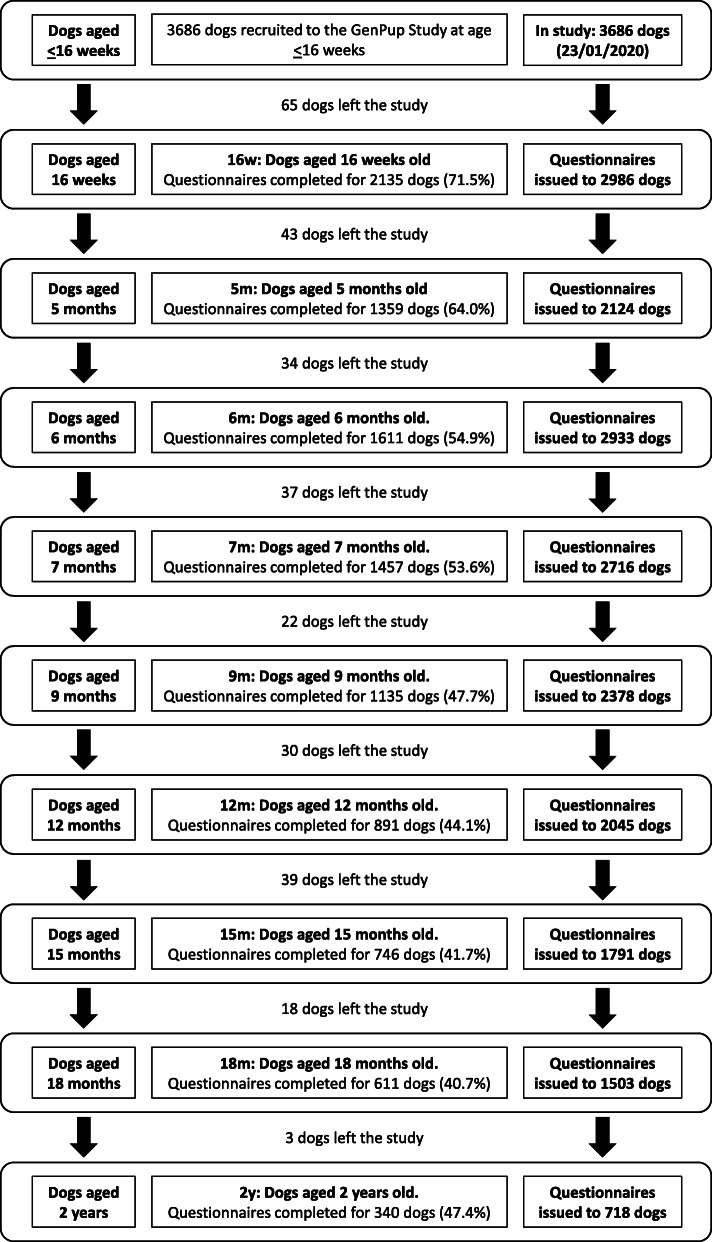
Numbers of questionnaires completed by owners of dogs registered on the Generation Pup Study. Legend: ^a^ Fig. 1 is based on 3686 dogs rather than 3727 dogs, as partially completed questionnaires and paper-based questionnaire data were not included. The two additional questionnaires issued to the ‘early cohort’ (*n*=200) are not included in this figure

Response rates were calculated from the proportion of questionnaires that were fully-completed once the questionnaire had reached its expiry date (Table 1), using the number of questionnaires available for completion as the denominator (i.e. excluding dogs that had been withdrawn from the study). For example, for the 12-month questionnaire, the number (%) of fully-completed questionnaires were calculated for dogs remaining in the study and aged at least 408 days. Figure 1 summarises the number of fully-completed questionnaires at each time point, and the associated response rate (%) for completion.

Most questionnaires that are started are completed, with part-completions accounting for 0.8–9.7% of all questionnaires issued at each timepoint. Part-completions are typically lower for shorter questionnaires (e.g. 0.8% for the 5 m questionnaire), and higher for early questionnaires (e.g. 9.7% for the 16 w questionnaire), potentially because the more committed owners are retained over time.

To date, a full set of questionnaires have been completed at early timepoints 16 w, 6 m, 7 m, 9 m, 12 m, (i.e. all timepoints from registration to age 12 months, excluding the 5 m survey that was not available to all dogs), for 31.6% (638/2045) of the cohort that were issued these questionnaires and had reached 407 days of age (the age at which the 12-month questionnaire can no longer be completed).

Table 4 details the number and percentage of owners providing consent for, and submitting ‘vet cards’, canine biological samples and signed consent forms for access to clinical veterinary notes by February 2020.

**Table 4:** Consent to access clinical notes, consent and submission of veterinary health cards and biological samples

	Provided consent	Submitted (12-16wks)	Submitted (6 m)	Submitted (12 m)	Submitted (2y)
	N (%) =entire cohort	N (%) =from mailout	N (%) =from mailout	N (%) =from mailout	N (%) =from mailout
**Veterinary Health Cards**^**a**^					
**Oral Health (OH) Card**^**b**^	3041/3555 (85.5%)	n/a	n/a	238/1392 (17.1%)	101/797 (12.7%)
**Heart Murmur (HM) Card**^**b**^	3041/3555 (85.5%)	792/3098 (25.6%)	n/a	244/1392 (17.5%)	104/797 (13.0%)
**Body Condition Score (BCS) Card**^**c**^	3041/3555 (85.5%)	713/3098 (23.0%)	n/a	233/1392 (16.7%)	103/797 (12.9%)
**Canine Biological Samples (Collected and submitted by owners)**^**d**^					
**Hair brushings**^**e**^	3404/3720 (91.5)	1462/3072 (47.6)	n/a	366/1394 (26.3)	266/858 (31.0)
**Skin swab**^**e**^	3104/3720 (83.4)	n/a	348/903 (38.5)	283/1280 (22.1)	223/792 (28.2)
**Buccal swab**^**e**^	3345/3720 (89.9)	1419/3010 (46.3)	n/a	378/1375 (27.5)	n/a
**Urine**^**e**^	2578/3720 (69.3)	954/2309 (41.3)	n/a	289/1075 (26.9)	147/661 (22.2)
**Faeces**^**e**^	2989/3720 (80.3)	1293/2690 (48.1)	351/870 (40.3)	410/1236 (33.2)	214/768 (27.9)
**Clinical records**	3053/3720 (82.1%)	1359/3720 (36.5%) - all ages			

^a^Owners of 171 dogs from the ‘early cohort’ were not asked for their consent to participate in the ‘vet health card’ aspect of the study

^b^Completed by veterinarians

^c^Completed by veterinarians or veterinary nurses

^d^Missing data for consent to participate in the ‘canine biological samples’ aspect of the study for owners of 6 dogs

^e^Samples submitted by 14/2/2020

Despite strategies to maintain owner-engagement with the study, inevitably some dogs will be lost to the study, for example due to mortality. The most common reasons given by owners for removing their dogs (*n*=281) from the study to date (23/1/2020) included lack of time (34.5%), the dog had been rehomed/relinquished (14.9%) or the dog had died/been euthanised (8.5%). Owners also reported other reasons for leaving the study, which are described in Table 5. Current data (Table 5) indicates a loss to follow up rate of 10.2% (231/(2065+ 22+ 37+ 77+ 65)) to age 12 m and 7.7% (60/(721+ 18+ 39)) between age 12 m and 2y.

**Table 5:** Loss to follow up between completion of the ‘About my puppy’ and the ‘two-year’ questionnaires

Timepoints (numberof dogs	Number of dogs leaving the study (% of dogs leaving between these timepoints, based on the denominator of thenumber of dogs who have, or would have, reached the age of the upper timepoint)					
	Lack of time	Dog died / euthanised	Dog rehomed orsurrendered to ananimal rehomingorganization	Would rather not say/ no information given	Other^**a**^	Total number of dogsleaving the studybetween timepoints.(% of dogs)
**AMP-16w**	19 (29.2)	3 (4.6)	7 (10.8)	30 (46.2)	6 (9.2)	65 / 3051 (2.1)
**16w-6m**^**b**^	32 (41.6)	6 (7.8)	6 (7.8)	30 (39.0)	3 (3.9)	77 / 3010 (2.6)
**6 m–7 m**	11 (29.7)	3 (8.1)	13 (35.1)	10 (27.0)	0 (0.0)	37 / 2753 (1.3)
**7 m–9 m**	10 (45.5)	0 (0.0)	4 (18.2)	7 (31.8)	1 (4.5)	22 / 2400 (0.9)
**9 m–12 m**	7 (23.3)	3 (10.0)	7 (23.3)	12 (40.0)	1 (3.3)	30 / 2065 (1.4)
**12 m–15 m**	17 (43.6)	4 (10.3)	2 (5.1)	14 (35.9)	2 (5.1)	39 / 1830 (2.1)
**15 m–18 m**	6 (33.3)	4 (22.2)	3 (16.7)	3 (16.7)	2 (11.1)	18 / 1521 (1.2)
**18 m-2y**	1 (33.3)	1 (33.3)	0 (0.0)	1 (33.3)	0 (0.0)	3 / 721 (0.4)
***TOTAL***	***97 (34.5)***	***24 (8.5)***	***42 (14.9)***	***104 (37.0)***	***14 (5.0)***	***281***

^a^Other reasons included personal circumstances / dog and/or family member is ill / IT issues, e.g. no laptop, dogs were signed up in error as too old

^b^The 5 m questionnaire was not issued to all respondents who completed the 6 m questionnaire, hence 16w-5 m and 5 m–6 m data have been combined

Examination of data available for 2191 dogs who were/would have been 407 days old (the oldest age at which the 12 m questionnaire can be completed) between 16 April 2018 (the date of the 12 m survey launch) and 22 January 2020, revealed that the 12 m questionnaire had been partially (*n*=167) or fully completed (*n*=896) for 1063 of these dogs. Thus, including owners who had not received notification of their 12 m questionnaire – due to their dogs already having been removed from the study (*n*=144), and owners who had not started their 12 m questionnaire despite their dogs still being registered with the study (*n*= 983), 48.5% of owners who had registered a puppy with the study provided questionnaire data when their dogs were aged 12-months.

#### Methodological implications of the Covid-19 pandemic

Canine biological sampling packs and veterinary health cards have not been mailed to participants since 17 March 2020, due to restrictions in place regarding access to office space and temporary closure of laboratories for processing biological samples. Mailouts of veterinary health cards and canine biological sampling packs resumed in July 2020 and October 2020, respectively. 

## Discussion

The Generation Pup project is the first longitudinal study to investigate both health and behaviour outcomes in a large cohort of dogs throughout their lives. The inclusion of all breeds and mixed-breed dogs will enable breed differences to be studied for outcomes, subject to sample size for specific breeds and outcomes.

No national register of dogs in the UK exists which could be used to assess representativeness of dogs and owners registering for the Generation Pup study; however, it is recognised that the cohort is likely to suffer from sampling bias due to self-selection by owners.

Currently, 65.7% (2446/3723) of the cohort are described by their owners as being a ‘specific named breed’, compared with 77.9% of dogs reported as pure-bred in a study of 5095 dogs registered with first opinion veterinary practices within England [11], 73.8% of a sample of insured dogs [3] and 64.0% of micro-chipped registrations [3]. It should be noted that comparative data were obtained prior to the end of 2011 [3, 11] and more up-to-date figures are needed to provide a true comparison reflecting the growing popularity of ‘designer cross-breeds’ [45]. Comparisons of the breeds most commonly registered with Generation Pup and/or with the UK Kennel Club, revealed that the proportion of dogs of specific breeds registered to date with the Generation Pup study are broadly similar to the proportion of these breeds registered with the Kennel Club (Table 3), with the exception of French Bulldogs, Pugs and Bulldogs that are currently under-represented and Border Collies, Golden Retrievers, Jack Russell Terriers and Flat Coated Retrievers that are over-represented within our dataset, when compared to UK Kennel Club data. It is recognised that not all ‘specific named breed’ dogs are registered with the Kennel Club, as shown by our data. To date, at the time of registration with the study, of 2446 dogs reported as ‘specific named breeds’, 33.0% (808/2446) were reportedly not registered with the Kennel Club, and owners of a further 3.7% (90/2446) dogs did not know if they were registered with the Kennel Club. Previous research noted that some breeds, including Greyhounds, Border Collies, Dalmatians, Rottweilers and Yorkshire Terriers were under-reported in Kennel Club registrations, compared with 2008 micro-chip registrations, whilst others were over-represented within the Kennel Club dataset, including Pugs and Dachshunds [3]. Targeting recruitment of under-represented breeds according to Kennel Club statistics, and other data sources that become available, will be prioritised in order to increase the sample size and thus the opportunity to include these popular breeds as covariates in future analyses.

As anticipated, the overwhelming majority (89.7%) of dog owners participating in the Generation Pup study are female. In comparison, of respondents participating in a telephone survey about pet ownership, 66.6% of respondents who owned a dog were female, compared with 60.1% of respondents who did not own a dog [46]. Similar to other longitudinal studies, this project may be less able to attract and retain participants from lower socio-economic backgrounds [47]. In the absence of National data relating to demographics of dog owners, the profile of Generation Pup participants to date (Table 2) was compared with the profiles of dog-owning respondents in a telephone survey about ownership of cats and dogs within the UK [46] and dog-owning residents interviewed in a community in Cheshire, UK [48] (Additional file 4). To date, owners participating in the Generation Pup study are less likely to be aged > 55 years, to live in urban/semi-urban locations, to live in a household where the highest level of qualification is at A levels or less compared with owners participating in these two other surveys of dog ownership [46, 48].

Bias resulting from differential selection may have implications on some exposure and outcome measures (e.g. dog management practices, such as preventative health care, training and socialisation). Consequently, prevalence estimates from a longitudinal study should be treated with caution and factors related to exposures and selection should be controlled for in analyses [49]. Despite potential selection bias within our cohort, previous work has suggested prioritising enrolling motivated participants who are more likely to retain their involvement over time over representativeness of the cohort compared to the target population, as the effect of selection bias on exposure-outcome associations was limited [47].

In contrast to studies based solely on data obtained from veterinary records, the Generation Pup project is able to include in risk factor analyses, detailed owner-reported data relating to the management of the dog and household/environmental factors. Owner-reports of clinical signs enable the investigation of canine health problems that are either not presented for veterinary investigation, or only presented once the clinical signs become more chronic or serious in nature. Whilst owner-reported data of clinical signs must be treated with caution, comparison of owner reports with data provided by veterinary practices will be conducted where possible to assess for bias. Analysis of owner-reported canine health data has the potential to identify factors that can be targeted to provide early intervention of problems.

The Generation Pup project is unique and ambitious. In addition to collecting owner-reported canine health data, behaviour data are collected via detailed questions that are repeated across different contexts and at different timepoints. Whilst behaviourists and researchers appreciate the need for repeated/similar questions, it might be challenging to maintain the enthusiasm of dog owners over time with respect to this point. A challenge of any long-term longitudinal study is to maintain engagement of participants over time and to reduce bias associated with differential loss to follow up. Loss to follow up within our study has been quantified at this relatively early stage. At age 12-months, owners of 48.5% (1063/2192) dogs were still engaged in the study (i.e. they had not withdrawn from the study and had started or completed their 12 m questionnaire). Ongoing participation rates in the Generation Pup study are comparable with rates reported from the longitudinal UK study of Labrador Retriever dogs, where 39–45% of owners were reported to be actively involved in the study when dogs were aged 400 days or more [23].

Future work will include investigation of factors associated with loss to follow up for all areas of data collection, (including questionnaires, vet cards, access to veterinary clinical notes, biological samples) within our cohort.

Enquiries regarding potential collaboration on areas of analysis and/or access to data for research purposes to benefit canine welfare can be made by contacting generationpup@dogstrust.org.uk.

## Conclusions

The Generation Pup project is a ground-breaking study of canine health and behaviour with enormous potential to identify associations and interactions between management including early-life environment, genotypic make-up and outcomes at different life stages. Modifiable risk factors can be used to improve canine health and welfare.

## Supplementary Information

**Additional file 1.** Questionnaires that have been developed to date (27 May 2020) for the Generation Pup study. Step titles and links to PDF copies of questionnaires are included.

**Additional file 2.** Veterinary health cards.

**Additional file 3.** Power Calculations.

**Additional file 4.** Comparison of Generation Pup owner-reported demographic data with data reported in two other studies.

**Additional file 5.** Consent forms.

**Additional file 6.** Veterinary Consent form.

## Data Availability

The datasets generated and analysed during the current study are not publicly available due to ethical constraints but are available from the corresponding author or from generationpup@dogstrust.org.uk on reasonable request. Data shared will be pseudonymised so that no individual dogs or owners can be identified.

## Abbreviations

ALSPAC: Avon Longitudinal Study of Parents and Children
BCS: Body Condition Score
EC: Early cohort
HM: Heart Murmur
OH: Oral Health
OR: Odds Ratio
RoI: Republic of Ireland
UK: United Kingdom
UoB: University of Bristol
