# Supplementary material for: 'Generation Pup' – protocol for a longitudinal study of dog behaviour and health
Source: BMC Vet Res. 2021 Jan 4;17:1. doi: 10.1186/s12917-020-02730-8 (PMC7781182; doi:10.1186/s12917-020-02730-8)
Supplement: Supplementary file 1 — Additional file 1. Questionnaires that have been developed to date (27 May 2020) for the Generation Pup study. Step titles and links to PDF copies of questionnaires are included. [file 12917_2020_2730_MOESM1_ESM.pdf]

Additional file 1.

Questionnaires that have been developed to date (27 May 2020) for the Generation Pup study. Step titles and links to PDF copies of questionnaires are included.

**Questionnaire**<br>**Link to PDF copy of questionnaire**	**Step title**
About Me (AM)<br><br>https://generationpup.ac.uk/wp-content/uploads/2020/07/About-Me-questionnaire.pdf	1=My contact preferences<br>2=About me<br>3=My involvement in Generation Pup
About My Household (AMH)<br><br>https://generationpup.ac.uk/wp-content/uploads/2020/07/About-My-Household-questionnaire.pdf	1=Occupants<br>2=House<br>3=Health<br>4=Lifestyle<br>5=Education<br>6=Animals
About My Puppy (AMP)<br><br>https://generationpup.ac.uk/wp-content/uploads/2020/07/About-my-Puppy-questionnaire.pdf	1=My puppy<br>2=My puppy’s breed and coat colour<br>3=My puppy’s documents<br>4=Whorls on my puppy’s coat<br>5=Choosing my puppy<br>6=Coming home<br>7=My puppy’s tail and dew claws<br>8=Plans for my puppy<br>9=My plans for neutering my puppy
Settling in (SI)<br><br>https://generationpup.ac.uk/wp-content/uploads/2020/07/Settling-In-questionnaire.pdf	1=Introducing my puppy to my household<br>2=My puppy’s experiences<br>3=Meeting people<br>4=Meeting dogs from outside the household<br>5=My puppy’s behaviour<br>6=My puppy’s day<br>7=My puppy’s sleep<br>8=My puppy’s diet<br>9=My puppy’s training<br>10=My puppy’s health<br>11=Neuter status<br>12=Insurance status<br>13=Other information

12 weeks (12w)<br><br>https://generationpup.ac.uk/wp-content/uploads/2020/07/12-week-questionnaire.pdf	1=Introducing my puppy to my household<br>2=My puppy's experiences<br>3=Meeting other people<br>4=Meeting dogs from outside the household<br>5=My puppy's behaviour<br>6=My puppy's day<br>7=My puppy's sleep<br>8=My puppy's diet<br>9=My puppy's play<br>10=My puppy's training<br>11=My puppy's toileting<br>12=My puppy's classes<br>13=Training approaches<br>14=My puppy's health<br>15=Neuter status<br>16=Insurance<br>17=Other information
16 weeks (16w)<br><br>https://generationpup.ac.uk/wp-content/uploads/2020/07/16-week-questionnaire.pdf	1=Introducing my puppy to my household<br>2=My puppy's experiences<br>3=Meeting other people<br>4=Meeting dogs from outside the household<br>5=My puppy's behaviour<br>6=My puppy's day<br>7=My puppy's sleep<br>8=My puppy's diet<br>9=My puppy's play<br>10=My puppy's exercise away from home<br>11=My puppy's training<br>12=My puppy's toileting<br>13=My puppy's classes<br>14=Training approaches<br>15=My puppy's behavioural development<br>16=My puppy's health<br>17=Neuter status<br>18=Insurance<br>19=Other information
5 months (5m)<br><br>https://generationpup.ac.uk/wp-content/uploads/2020/07/5-months-questionnaire.pdf	1=How is your puppy?<br>2=Mobility and exercise
6 months (6m)<br><br>https://generationpup.ac.uk/wp-content/uploads/2020/07/6-months-questionnaire.pdf	1=Meeting other people<br>2=Meeting other dogs<br>3=My puppy's experiences<br>4=My puppy's boarding/kenneling experience<br>5=My puppy's day<br>6=My puppy's health<br>7=My puppy's behaviour<br>8=Insurance Status<br>9=Other information

7 months (7m)<br><br>https://generationpup.ac.uk/wp-content/uploads/2020/07/7-months-questionnaire.pdf	1=My puppy's diet<br>2=My puppy's training and toileting<br>3=My puppy's recent behaviour<br>4=My puppy's exercise away from home<br>5=Neuter status<br>6=Household environment<br>7=Other information
9 months (9m)<br><br>https://generationpup.ac.uk/wp-content/uploads/2020/07/9-month-questionnaire.pdf	1=My puppy's health<br>2=Neutering, surgery and breeding<br>3=My puppy's behaviour<br>4=My puppy's training<br>5=My puppy's boarding/kenneling experience and sleep<br>6=My puppy's day<br>7=My puppy's experiences<br>8=Meeting other people<br>9=Meeting other dogs<br>10=My puppy's exercise away from home<br>11=My puppy's diet<br>12=Other information

12 months (12m)<br><br>https://generationpup.ac.uk/wp-content/uploads/2020/07/12-month-questionnaire.pdf	1=In the house and ‘out and about’<br>2=My dog’s behaviour<br>3=My dog’s recent behaviour<br>4=My dog’s training<br>5=My dog’s health<br>6=Breeding<br>7=My dog’s boarding/kenneling experience and sleep<br>8=My dog’s diet<br>9=My dog’s day<br>10=My dog’s experiences<br>11=Meeting people<br>12=Meeting other dogs<br>13=Miscellaneous<br>14=Neutering<br>15=Surgery<br>16=Further information
15 months (15m)<br><br>https://generationpup.ac.uk/wp-content/uploads/2020/07/15-month-questionnaire.pdf	1=Dogs and cats in the household<br>2=My dog’s play<br>3=My dog’s exercise away from home<br>4=My dog’s training<br>5=My dog’s behaviour<br>6=My dog’s diet<br>7=Health Scores and insurance *(thereafter available on dashboard for updated data entry)*<br>8=My dog’s health<br>9=Neutering<br>10=Surgery<br>11=Breeding<br>12=Further information

18 months (18m)<br><br>https://generationpup.ac.uk/wp-content/uploads/2020/07/18-month-questionnaire.pdf	1=My dog’s boarding/kenneling experience and sleep<br>2=My dog’s health<br>3=Neutering<br>4=Surgery<br>5=Breeding<br>6=Mobility and Exercise<br>7=My dog’s diet<br>8=My dog’s eating behaviour<br>9=My dog’s behaviour<br>10=My dog’s exercise away from home<br>11=My dog’s activities<br>12=My dog’s day<br>13=Meeting people<br>14=Meeting other dogs<br>15=Further information
2 years (2y)<br><br>https://generationpup.ac.uk/wp-content/uploads/2020/07/2-year-questionnaire.pdf	1=Reflections<br>2=My dog’s health<br>3=In the house and ‘out and about’<br>4=Neutering<br>5=Surgery<br>6=Breeding<br>7=My dog’s play<br>8=My dog’s exercise away from home<br>9=My dog’s training<br>10=Behaviour<br>11=Miscellaneous<br>12=Further information

2.5 years (2.5y)<br><br>https://generationpup.ac.uk/wp-content/uploads/2020/07/2.5-year-questionnaire.pdf	1=My dog’s health<br>2=My dog’s neutering<br>3=My dog’s surgery<br>4=Mobility<br>5=Insurance<br>6=Meeting people<br>7=Meeting other dogs<br>8=My dog’s exercise away from home<br>9=My dog’s activities<br>10=My dog’s day<br>11=My dog’s sleep<br>12=My dog’s behaviour<br>13=My dog’s diet<br>14=My dog’s eating behaviour<br>15=My dog’s experiences<br>16=Further information
3 years (3y)<br><br>https://generationpup.ac.uk/wp-content/uploads/2020/07/3-year-questionnaire.pdf	1=My dog’s health<br>2=In the house and ‘out and about’<br>3=Neutering<br>4=Surgery<br>5=Breeding<br>6=My dog’s play<br>7=My dog’s exercise away from home<br>8=My dog’s training<br>9=Behaviour<br>10=Miscellaneous<br>11=Further information

3.5 years (3.5y)<br><br>https://generationpup.ac.uk/wp-content/uploads/2020/07/3.5-year-questionnaire.pdf	1=My dog's health<br>2=My dog's neutering<br>3=My dog's surgery<br>4=Mobility<br>5=Insurance<br>6=Meeting people<br>7=Meeting other dogs<br>8=My dog's exercise away from home<br>9=My dog's activities<br>10=My dog's day<br>11=My dog's sleep<br>12=My dog's behaviour<br>13=My dog's diet<br>14=My dog's eating behaviour<br>15=My dog's experiences<br>16=Further information
4 years (4y)<br><br>https://generationpup.ac.uk/wp-content/uploads/2020/07/4-year-questionnaire.pdf	1=Reflections<br>2=My dog's health<br>3=In the house and 'out and about'<br>4=Neutering<br>5=Surgery<br>6=Breeding<br>7=My dog's play<br>8=My dog's exercise away from home<br>9=My dog's training<br>10=Behaviour<br>11=Miscellaneous<br>12=Further information
Catch up Survey (September 2017)<br><br>https://generationpup.ac.uk/wp-content/uploads/2020/07/Generation-Pup-cohort-profile-Additional-file-2-Catch-Up-Survey.pdf	1=My household<br>2=Meeting people<br>3=Meeting dogs<br>4=My dog's diet<br>5=My dog's behavior<br>6=My dog's health<br>7=Neuter status<br>8=Other information
