# Supplementary material for: 'Generation Pup' – protocol for a longitudinal study of dog behaviour and health
Source: BMC Vet Res. 2021 Jan 4;17:1. doi: 10.1186/s12917-020-02730-8 (PMC7781182; doi:10.1186/s12917-020-02730-8)
Supplement: Supplementary file 2 — Additional file 2. Veterinary health cards. [file 12917_2020_2730_MOESM2_ESM.pdf]

Additional file 2

Oral health card

Side 1:

Information for Dog Owner

Dear Generation Pup participant,

Please take this oral health card with you when you take your dog to visit your vet practice. If your dog has an annual check-up or vaccinations, that could be a convenient time. Please ask your vet to fill it in. After your appointment, please return this card to us in the prepaid envelope provided. Alternatively, you can scan or take a photo and email it to generationpup@dogstrust.org.uk

We appreciate your time and commitment and are grateful that you have agreed to help us with the 'Vet Cards' aspect of our study. Thank you.

Best wishes,
The Generation Pup team.

We are hoping to access veterinary records (with owner permission) in the future. It would help us if you could add the text GENPUP ID (followed by this dog's unique Generation Pup Dog ID number found on the front of this card) into this dog's notes please.

Information for Vet

Dear Practice Colleague,

Please complete this card during this dog's visit. This dog is participating in Generation Pup, which is a clinical research project funded by Dogs Trust and run by researchers at Dogs Trust and the University of Bristol. Generation Pup aims to be the most comprehensive study of dog health, behaviour and welfare in a generation by using a longitudinal study of all breeds and cross-breeds of dogs from across the UK and Republic of Ireland.

For more information, please visit our website: https://generationpup.ac.uk.
Thank you for your time.

Best wishes,
The Generation Pup team.

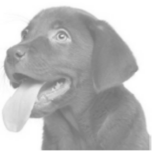

Generation PUP

DogsTrust Reg charity no's: 227523 & SC037843

University of BRISTOL

Side 2:

Generation Pup Oral Health Card for your vet

Generation PUP

Using the diagram below, please score the **six marked teeth on the left-side of the dog's mouth** using the scales provided.

Tooth	Calculus grade	Gingivitis grade
Upper I3		
Upper C		
Upper P4		
Lower I3		
Lower C		
Lower P4		

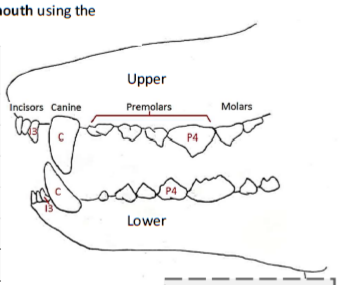

Vet practice stamp:

Date: ___/___/___

CALCULUS SCALE

0 – No calculus (but tooth present)
1 – Scant areas of calculus (not forming a band)
2 – A band of calculus covering less than one-third of the tooth
3 – A band of calculus covering one-third to two-thirds of the tooth
4 – Calculus covering more than two-thirds of the tooth
M – Missing tooth

GINGIVITIS SCALE

0 – No inflammation
1 – Mild inflammation (slight change in colour, thin red line)
2 – Moderate inflammation (thick red line or some glazing, redness, oedema of the entire gum)
3 – Severe inflammation (redness, glazing of the entire gum)

Please use the space below to make additional notes about the general dentition (e.g. crowding, fractured/chipped/discoloured/missing teeth, gum recession or other tooth abnormalities), and if possible/appropriate describe which teeth are affected.

Notes: ______________________________

Heart Murmur card

Side 1:

Information for Dog Owner

Dear Generation Pup participant,

Please take this heart murmur card with you when you next take your dog to visit your vet and ask your vet to fill it in for you. After your appointment, please return this card to us in the prepaid envelope provided. Alternatively, you can scan or take a photo and email it to generationpup@dogstrust.org.uk.

Thank you ***very much*** for being part of the Generation Pup study. We are grateful that you have agreed to help us with the 'Vet Cards' aspect of our study.

Best wishes,
The Generation Pup team.

Information for Vet

We are hoping to access veterinary records (with owner permission) in the future. It would help us if you could add the text GENPUP ID (followed by this dog's unique Generation Pup Dog ID number found on the front of this card) into this dog's notes please.

Dear Colleague,

We would be grateful if you could please complete this card during this dog owner's visit.

Generation Pup is a clinical research project run by researchers at Dogs Trust and the University of Bristol. Generation Pup aims to be the most comprehensive study of dog health, behaviour and welfare in a generation by using a longitudinal study of all breeds and cross-breeds of dogs from across the UK and Republic of Ireland. For more information, please visit http://generationpup.ac.uk. Thank you for your time.

Best wishes,
The Generation Pup team.

Generation PUP

DogsTrust
Reg charity no's: 227523 & SC037843

University of BRISTOL

Side 2:

Heart Murmur Card for your vet to complete

Generation PUP

The dog's...

Heart rate _____ /min   Respiratory rate _____ /min

The dog's pulse quality is... *(Tick one)*

Weak	Adequate	Strong
☐	☐	☐

The dog's heart rhythm is... *(Tick one)*

Regular (include sinus arrhythmia here)	Irregular
☐	☐

The dog has a heart murmur... *(Tick one)*

Yes ☐   No ☐

IF YES

Vet practice stamp:

Date: ___/___/___

The murmur is... *(Tick one)*

Quieter than the normal heart sounds	As loud as the normal heart sounds	Louder than the normal heart sounds
☐	☐	☐

The murmur can be heard louder on the... *(Tick all that apply)*

Right side of the chest	Left side of the chest	Equal on both right and left sides
☐	☐	☐

The murmur can be heard louder... *(Tick one)*

At the heart base	At the heart apex	Not possible to localise
☐	☐	☐

The murmur is... *(Tick one)*

Diastolic	Systolic	Continuous
☐	☐	☐

Body Condition Score card

Side 1:

Information for Dog Owner

Dear Generation Pup participant,

Please take this body condition score card with you when you next take your dog to visit your vet. If your dog has an annual check-up or vaccinations, that could be a convenient time. Please ask your vet/vet nurse to fill it in for you. After your appointment, please return this card to us in the prepaid envelope provided. Alternatively, you can scan or take a photo and email it to generationpup@dogstrust.org.uk.

Thank you very much for being part of the Generation Pup study. We are grateful that you have agreed to help us with the 'Vet Cards' aspect of our study.

Best wishes,
The Generation Pup team.

Information for Vet/Vet nurse

We are hoping to access veterinary records (with owner permission) in the future. It would help us if you could add the text GENPUP ID (followed by this dog's unique Generation Pup Dog ID number found on the front of this card) into this dog's notes please.

Dear Practice Colleague,

We would be grateful if you could please complete this card and return it to the dog's owner during their visit. *Please ignore the references to the dog's abdominal tuck if the dog is less than 6 months of age.*

Generation Pup is a clinical research project run by researchers at Dogs Trust and the University of Bristol. Generation Pup aims to be the most comprehensive study of dog health, behaviour and welfare in a generation by using a longitudinal study of all breeds and cross-breeds of dogs from across the UK and Republic of Ireland. For more information, please visit http://generationpup.ac.uk. Thank you for your time.

Best wishes,
The Generation Pup team.

Generation PUP

DogsTrust Reg charity no's: 227523 & SC037843

University of BRISTOL

Side 2:

Body Condition Score Card for your Vet/Vet Nurse to complete

Vet practice stamp:

Date: ___/___/___

Using the below score chart, please rate this dog's body condition score.

This dog's body condition score is ____ / 9.

If you weighed this dog, please add the weight here ______ kgs.

1
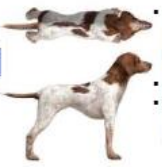

- Ribs, lumbar vertebrae, pelvic bones and all bony prominences evident from a distance
- No discernible body fat
- Obvious loss of muscle mass

2
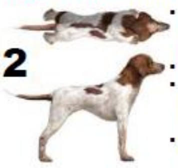

- Ribs, lumbar vertebrae, and pelvic bones easily visible
- No palpable fat
- Some bony prominences visible from a distance
- Minimal loss of muscle mass

3
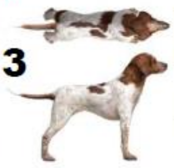

- Ribs easily palpable and may be visible with no palpable fat
- Tops of lumbar vertebrae visible, pelvic bones becoming prominent
- Obvious waist and abdominal tuck

4
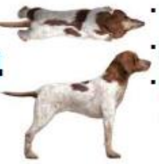

- Ribs easily palpable with minimal fat covering
- Waist easily noted when viewed from above
- Abdominal tuck evident

5
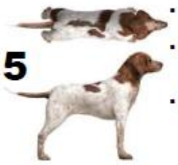

- Ribs palpable without excess fat covering
- Waist observed behind ribs when viewed from above
- Abdomen tucked up when viewed from side

6
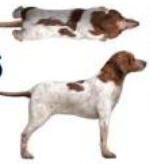

- Ribs palpable with slight excess of fat covering
- Waist is discernible when viewed from above but not prominent
- Abdominal tuck apparent

7
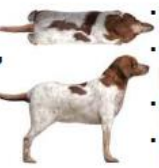

- Ribs palpable with difficulty, heavy fat cover
- Noticeable fat deposits over lumbar area and base of tail
- Waist absent or barely visible
- Abdominal tuck may be absent

8
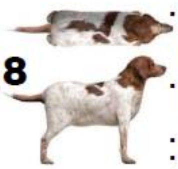

- Ribs not palpable under very heavy fat cover or palpable only with significant pressure
- Heavy fat deposits over lumbar area and base of tail
- Waist absent
- No abdominal tuck
- Obvious abdominal distension may be present

9
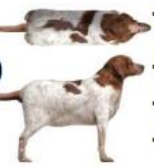

- Massive fat deposits over thorax, spine, and base of tail
- Waist and abdominal tuck absent
- Fat deposits on neck and limbs
- Obvious abdominal distension

© ROYAL CANIN SAS 2014 - All rights reserved. Adapted from Laflamme DP. Development and validation of a body condition score system for dogs. Canine practice vol 22, N° 4, 1997 Code:18817
