# Supplementary material for: 'Generation Pup' – protocol for a longitudinal study of dog behaviour and health
Source: BMC Vet Res. 2021 Jan 4;17:1. doi: 10.1186/s12917-020-02730-8 (PMC7781182; doi:10.1186/s12917-020-02730-8)
Supplement: Supplementary file 3 — Additional file 3. Power Calculations. [file 12917_2020_2730_MOESM3_ESM.pdf]

Additional file 3

Power calculations for the Generation Pup study.

Age of dogs (years)	Number of dogs estimated to leave the study, during the previous 12 months[a]	Number of dogs estimated to remain in the study	Number of dogs estimated to have data available for analysis, allowing for missing data [b]	Minimum Prevalence to detect OR≥1.50 [c]<br>30%<br>20%<br>10%	Minimum Prevalence to detect OR≥1.75 [c]<br>30%<br>20%<br>10%	Minimum Prevalence to detect OR≥2.00 [c]<br>30%<br>20%<br>10%
**Total number of dogs in cohort = 10,000**						
1	1020	8980	3143	8.9<br>11.1<br>18.7	4.6<br>5.6<br>9.2	2.9<br>3.5<br>5.7
2	691	8289	2901	9.7<br>12.0<br>20.3	5.0<br>6.0<br>10.0	3.2<br>3.8<br>6.2
3	497	7791	2727	10.3<br>12.8<br>21.6	5.3<br>6.4<br>10.6	3.4<br>4.1<br>6.6
4	467	7324	2563	10.9<br>13.6<br>22.9	5.6<br>6.8<br>11.3	3.6<br>4.3<br>7.0
5	439	6884	2410	11.6<br>14.5<br>24.4	6.0<br>7.3<br>12.0	3.8<br>4.6<br>7.4
6	413	6471	2265	12.4<br>15.4<br>26.0	6.4<br>7.7<br>12.8	4.1<br>4.9<br>7.9
7	388	6083	2129	13.2<br>16.4<br>27.6	6.8<br>8.2<br>13.6	4.3<br>5.2<br>8.4
8	365	5718	2001	14.0<br>17.4<br>29.4	7.2<br>8.7<br>14.4	4.6<br>5.6<br>8.9
9	343	5375	1881	14.9<br>18.6<br>31.3	7.7<br>9.3<br>15.4	4.9<br>5.9<br>9.5
10	322	5052	1768	15.8<br>19.7<br>33.3	8.1<br>9.9<br>16.3	5.2<br>6.3<br>10.1
11	303	4749	1662	16.8<br>21.0<br>35.4	8.7<br>10.5<br>17.4	5.5<br>6.7<br>10.8
12	285	4464	1563	17.9<br>22.3<br>37.6	9.2<br>11.2<br>18.5	5.9<br>7.1<br>11.5

[a] Based on conservative loss to follow up rates based on current data (Table 6) of 10.2% (231/2266) to age 12m and 7.7% (60/778) between age 12m and 2y and

assuming a reduced LTFU thereafter of 6% annually (based on reduced numbers of owners leaving the study through being disengaged, but with higher numbers of mortality as dogs age).
[b] The number of dogs that could potentially be included in analyses for outcome data at different age timepoints has been calculated, assuming that 35% of remaining dogs have a complete dataset available for analysis.
[c] http://sampsize.sourceforge.net/iface/s3.html#cc
