# Supplementary material for: 'Generation Pup' – protocol for a longitudinal study of dog behaviour and health
Source: BMC Vet Res. 2021 Jan 4;17:1. doi: 10.1186/s12917-020-02730-8 (PMC7781182; doi:10.1186/s12917-020-02730-8)
Supplement: Supplementary file 4 — Additional file 4. Comparison of Generation Pup owner-reported demographic data with data reported in two other studies. [file 12917_2020_2730_MOESM4_ESM.pdf]

Additional file 4.

Comparison of Generation Pup owner-reported demographic data with data reported in two other studies.

	Generation Pup	Murray et al. 2010[a]	Westgarth et al.[b]
Household – single adult, no children	597/3722<br>16.0% (95%CI 14.8-17.2)	129/784<br>16.5% (95%CI 13.9-19.1	39/261<br>14.9% (95%CI 10.6-19.2)
Cat(s) in household	1034/3722<br>27.8% (95%CI 26.4-29.2)	191/784<br>24.4% (95%CI 21.4-27.4)	
Urban/semi-urban location	1577/3704<br>42.6% (95%CI 41.0-44.2)	519/784<br>66.2% (95%CI 62.9-69.5)	
Highest level of qualification achieved within household: A level or less	880/3722<br>23.6% (95%CI 22.2-25.0)	483/784<br>61.6% (95%CI 58.2-65.0)	
Age of respondent: ≥55 years	876/3715<br>23.6% (95%CI 22.2-25.0)	285/784<br>36.4% (95%CI 33.0-39.8)	
Retired	Respondent:<br>447/3649<br>12.2% (95%CI 11.1-13.3)		Person within household:<br>67/260<br>25.8% (95% CI 20.5-31.1)
Unemployed	155/3649<br>4.2% (95% CI 3.6-4.9)		Person within household:<br>8/260<br>3.1% (95% CI 1.0-5.2)

[a] Murray J, Browne W, Roberts M, Whitmarsh A, Gruffydd-Jones T. Number and ownership profiles of cats and dogs in the UK. Vet Rec 2010; 166:163-8.

[b] Westgarth C, Pinchbeck GL, Bradshaw JWS, Dawson S, Gaskell RM, Christley RM. Factors associated with dog ownership and contact with dogs in a UK community. BMC Vet Res, 2007; doi: 10.1186/1746-6148-3-5.
