# Supplementary material for: 'Generation Pup' – protocol for a longitudinal study of dog behaviour and health
Source: BMC Vet Res. 2021 Jan 4;17:1. doi: 10.1186/s12917-020-02730-8 (PMC7781182; doi:10.1186/s12917-020-02730-8)
Supplement: Supplementary file 5 — Additional file 5. Consent forms. [file 12917_2020_2730_MOESM5_ESM.pdf]

GENERATION PUP REGISTRATION FORM

YOUR INFORMATION

Title:	First name:	Surname:	Gender:<br>☐ M ☐ F
Address (required):			
Postcode/Eircode (required):			

Home phone number (optional):	Mobile phone number (optional):	Email address (optional):

PUPPY INFORMATION

Name of puppy 1:	Puppy's birth date:<br>DD / MM / YY	Date puppy joined your household:<br>DD / MM / YY	Puppy's gender:<br>☐ M ☐ F
Name of puppy 2:	Puppy's birth date:<br>DD / MM / YY	Date puppy joined your household:<br>DD / MM / YY	Puppy's gender:<br>☐ M ☐ F
Name of puppy 3:	Puppy's birth date:<br>DD / MM / YY	Date puppy joined your household:<br>DD / MM / YY	Puppy's gender:<br>☐ M ☐ F
Name of puppy 4:	Puppy's birth date:<br>DD / MM / YY	Date puppy joined your household:<br>DD / MM / YY	Puppy's gender:<br>☐ M ☐ F
Name of puppy 5:	Puppy's birth date:<br>DD / MM / YY	Date puppy joined your household:<br>DD / MM / YY	Puppy's gender:<br>☐ M ☐ F

INFORMED CONSENT

Please note that you will need to agree to the first three requirements in order to take part in Generation Pup. If your puppy is owned jointly (for example with other household members), please ensure that all owners agree with the responses you are giving to the following questions.

**REQUIRED: I confirm** that I would like to participate in the Generation Pup study, that I am 16 years of age or older, and live in the UK or Republic of Ireland. I have read the information provided about the study, and understand who to contact if I have questions about it. I understand that all information about me will be treated in the strictest confidence. The data will be anonymised and stored securely. I also understand that I can reduce my involvement or withdraw from the study at any time without prejudice.	☐ Yes ☐ No

**REQUIRED: I agree** for the Generation Pup team to get in touch with me through the contact details I have chosen to supply. I understand my contact details may be used, for example, to notify me when questionnaires are ready to complete, send me questionnaires and sampling kits by post or provide further information about the study, such as newsletters. I understand that I can update, remove my details or request that contact is terminated at any point:	☐ Yes ☐ No
**REQUIRED: I agree** for the information I provide to be used for the purposes of research into dog health, behaviour or welfare. I understand that this may include sharing anonymised information or any samples that I have agreed to be stored from my dog (i.e. in a form in which I cannot be identified) with other universities or research organisations for the purposes of carrying out research for the benefit of dogs. I understand that publications and results arising from the Generation Pup study will not enable individual dogs or owners to be identified. I understand that I may withdraw this permission at any time without prejudice:	☐ Yes ☐ No
**OPTIONAL:** We would also like to ask for your consent to contact you about helping with research projects linked to 'Generation Pup'. This might be, for example, studies where we ask you to film aspects of your dog's behaviour, or have a researcher call you to ask additional questions. If you have time and are interested in helping us further then please let us know: we are very grateful for any help that you can give us to make 'Generation Pup' a success. I agree to being contacted by the Generation Pup team about involvement in related projects.	☐ Yes ☐ No
**OPTIONAL:** We would also like to ask if you might be interested in helping us to promote the study through the media. We would be very grateful if you could tick this option if you are willing for us to contact you about your puppy potentially becoming a 'media star'. We understand that this is not for everyone, so this is entirely optional.	☐ Yes ☐ No

OPTIONAL: If you have provided us with your phone number, are you happy for us to contact you by text and/or phone, on the rare occasion that we might need to? ☐ **Text only** ☐ **Phone only** ☐ **Both** ☐ **Neither**

YOUR SIGNATURE

Your signature:	Print your name:	Date:
____________________________	____________________________	________________

Thank you very much! The success of this study depends entirely on dog owners like yourself, and we are so grateful for any contribution you can make to help us make a difference for dogs. If you have any questions about the study, please do not hesitate to contact us by e-mailing us at generationpup@dogstrust.org.uk or phoning us on +44 (0)7434 843460.

Dogs Trust generously fund Generation Pup, and the study has been approved by the University of Bristol Animal Welfare Ethical Research Board (UIN/18/052).

For over 50 years, we've promised to never put down a healthy dog. We keep our promises, and that includes treating your personal details with care. We will keep the information you provide us with safe and will only use it for research purposes, pseudonymising data prior to analysis and publication. We also promise to only ever share your data (excluding personally identifiable information such as contact details, dog's name) with researchers who we authorise to access data we hold. We won't contact you for any other purpose, unless you already receive communications from us. You can opt out of these or change your preferences at any time by contacting generationpup@dogstrust.org.uk or phoning +44 (0)7434 843460. For more information on this visit our privacy section, dogstrust.org.uk/privacy.

DogsTrust

University of BRISTOL

Reg charity no's: 227523 & SC037843
