# Supplementary material for: 'Generation Pup' – protocol for a longitudinal study of dog behaviour and health
Source: BMC Vet Res. 2021 Jan 4;17:1. doi: 10.1186/s12917-020-02730-8 (PMC7781182; doi:10.1186/s12917-020-02730-8)
Supplement: Supplementary file 6 — Additional file 6. Veterinary Consent form. [file 12917_2020_2730_MOESM6_ESM.pdf]

Admin use only:	
Owner's ID: «userid»	Dog's ID: «dogId»

GENERATION PUP
VETERINARY RECORDS CONSENT FORM

WHY WOULD WE LIKE TO ACCESS YOUR PUPPY'S VET RECORDS?

We would like to collect vital information about the preventative health care and the clinical health of your puppy. To do this we would really appreciate access to your puppy's veterinary records. This will help us considerably, as we will be able to see what tests your puppy has had and whether your vet has diagnosed particular conditions. We will also be able to look across all the dogs in the study and explore factors which may be important in the development of certain conditions.

With your permission, we can collect this information from your veterinary practice directly. You can withdraw access to your veterinary records at any time by notifying us. Please ask your veterinary practice to enter your dog's GENPUP ID number into your puppy's clinical notes, **e.g. GENPUP0101**. (You can find your dog's ID on your Dashboard homepage.)

YOUR INFORMATION (owner to complete)

Title:	First name:	Surname:
Address (required):		Postcode (if relevant):
Name of dog(s) and Generation Pup ID number(s):		

YOUR VETERINARY PRACTICE (owner to complete)

Name of practice:	
Address (required):<br><br>Postcode (if relevant):	☐ I confirm that I have asked my veterinary practice to add **GENPUP** and my **dog's ID number** to their clinical notes, e.g. GENPUP0101.
Practice Phone number:	Practice Email address:

INFORMED CONSENT (owner to complete)

I give my/our permission as the owner(s) of my/our puppy for the Generation Pup team to contact my veterinary practice, and for my veterinary practice to email PDF copies of my puppy's veterinary records to the Generation Pup team.	☐ Yes ☐ No
I give my/our permission as the owner(s) of my/our puppy for the Generation Pup team to share information I have provided, in order to help the Generation Pup Team, access my puppy's veterinary records through databases held by other research groups. *(More information may be found in 'Note 1' on the next page of this document)*	☐ Yes ☐ No
I give my/our permission as the owner(s) of my/our puppy for the Generation Pup team to email a PDF copy of this consent form to the veterinary practice listed above, as evidence of my consent for access to veterinary records.	☐ Yes ☐ No
I understand that I can update, remove my details or request to withdraw access to my veterinary records at any time:	☐ Yes

YOUR SIGNATURE (owner to complete)

Your signature:	Print your name:	Date:
		d d / m m / y y

Thank you very much! If you have any questions about the study, please contact us by phoning us on **07434 843460** or e-mailing us at generationpup@dogstrust.org.uk

Registered Charity Numbers: 227523 & SC037843

Note 1

Other research groups (for example based at UK Universities) are already working with some veterinary practices. These research groups collect information from veterinary practice databases and analyse this information with the aim of further veterinary knowledge that can be used to improve the health and welfare of animals in the future. If your veterinary practice is working with one of these research groups, then – with your permission – we would like to access your veterinary notes via these research groups, rather than directly from your veterinary practice – thus potentially saving the time of staff at your veterinary practice.

If you give us permission to share information that can be used to identify your puppy in databases held by other research groups (for example your Generation Pup unique ID number and microchip number), then this would be an enormous help. You will have the right to withdraw permission for us to access your veterinary records through databases held by other research groups at any time by notifying us.

For over 50 years, we've promised to never put down a healthy dog. We keep our promises, and that includes treating your personal details with care. We will keep the information you provide us with safe and will only use it for research purposes, pseudonymising data prior to analysis and publication. We also promise to only ever share your data (excluding personally identifiable information such as contact details, dog's name) with researchers who we authorise to access data we hold. We won't contact you for any other purpose, unless you already receive communications from us. You can opt out of these or change your preferences at any time by contacting generationpup@dogstrust.org.uk or phoning +44 (0)7434 843460. For more information on this visit our privacy section, dogstrust.org.uk/privacy.
